# Src-Mediated Phosphorylation of the Tyrosine Phosphatase PRL-3 Is Required for PRL-3 Promotion of Rho Activation, Motility and Invasion

**DOI:** 10.1371/journal.pone.0064309

**Published:** 2013-05-17

**Authors:** James J. Fiordalisi, Brian J. Dewar, Lee M. Graves, James P. Madigan, Adrienne D. Cox

**Affiliations:** 1 Department of Radiation Oncology, University of North Carolina at Chapel Hill, Chapel Hill, North Carolina, United States of America; 2 Curriculum in Toxicology, University of North Carolina at Chapel Hill, Chapel Hill, North Carolina, United States of America; 3 Department of Pharmacology, University of North Carolina at Chapel Hill, Chapel Hill, North Carolina, United States of America; 4 Curriculum in Genetics & Molecular Biology, University of North Carolina at Chapel Hill, Chapel Hill, North Carolina, United States of America; 5 Lineberger Comprehensive Cancer Center, University of North Carolina at Chapel Hill, Chapel Hill, North Carolina, United States of America; Seoul National University, Republic Of Korea

## Abstract

The metastasis-associated tyrosine phosphatase PRL-3/PTP4A is upregulated in numerous cancers, but the mechanisms modulating PRL-3 activity other than its expression levels have not been investigated. Here we report evidence for both Src-dependent tyrosine phosphorylation of PRL-3 and Src-mediated regulation of PRL-3 biological activities. We used structural mutants, pharmacological inhibitors and siRNA to demonstrate Src-dependent phosphorylation of endogenous PRL-3 in SW480 colon cancer cells. We also demonstrated that PRL-3 was not tyrosine phosphorylated in SYF mouse embryo fibroblasts deficient in Src, Yes and Fyn unless Src was re-expressed. Further, we show that platelet-derived growth factor (PDGF) can stimulate PRL-3 phosphorylation in a Src-dependent manner. Finally, we show that PRL-3-induced cell motility, Matrigel invasion and activation of the cytoskeleton-regulating small GTPase RhoC were abrogated in the presence of the phosphodeficient PRL-3 mutant Y53F, or by use of a Src inhibitor. Thus, PRL-3 requires the activity of a Src kinase, likely Src itself, to promote these cancer-associated phenotypes. Our data establish a model for the regulation of PRL-3 by Src that supports the possibility of their coordinate roles in signaling pathways promoting invasion and metastasis, and supports simultaneous use of novel molecularly targeted therapeutics directed at these proteins.

## Introduction

Extensive evidence has accumulated linking the putative tyrosine phosphatase PRL-3 with invasion and metastasis (reviewed in [1]–[3]). Although the precise roster of its substrates remains to be fully determined, it is clear that PRL-3 mediated biological activities require its phosphatase activity and its lipid modification by farnesylation, as mutation of the catalytic residue C104 or the prenylated CAAX motif abrogates these functions [4]–[8]. However, little is known concerning the mechanism(s) through which PRL-3 itself is regulated, and it has largely been assumed that both its biochemical and biological activity correlate solely with its expression. Numerous studies have examined relative PRL-3 expression levels in cancer versus normal cells or tissues [1]–[3]. Other studies have demonstrated, for example, control of PRL-3 transcription by p53 [9], Snail [10], or MEF2C [11]; PCBP1-mediated control of PRL-3 translation [12]; and FKBP38-mediated control of PRL-3 protein stability [13]. One possible mechanism of regulation other than expression levels is post-translational modification of PRL-3, such as phosphorylation. In support of this possibility, there is evidence for the regulation of tyrosine phosphatases downstream of tyrosine kinases, including the proto-oncogene Src. For example, low molecular weight protein tyrosine phosphatase (LMW-PTP) is phosphorylated by Src [14], as well as by Fyn and Lck [15], and the EphA8 receptor tyrosine kinase [16]. This modification affects both the catalytic activity of LMW-PTP [16]–[18] and its association with the adapter protein Grb2 [17]. Similarly, phosphorylation of PTP1B by the insulin receptor [19], [20] or c-Met receptor [21] affects its catalytic activity, and phosphorylation of SHP-1 and SHP-2 lead to increases in their catalytic activity [22] and signaling [23], [24]. Interestingly, PRL-1, which shares all six tyrosines in common with PRL-3, was shown to be phosphorylated by Src *in vitro* [25], suggesting that PRL phosphatases may also be regulated by Src family kinases.

We therefore set out to determine a possible role for Src-mediated phosphorylation in the regulation of PRL-3 activity. Whether PRL-3 is phosphorylated by Src kinases was not known, nor were the potential functional consequences of such phosphorylation to any PRL family member examined. Here we report that PRL-3 is phosphorylated both *in vitro* and *in vivo* downstream of Src, and that this phosphorylation is required for PRL-3 to promote invasion, motility and activation of RhoC in cell culture systems. We also show that PRL-3 phosphorylation is stimulated by PDGF in a Src-dependent manner. Together these observations support a model in which Src, and possibly members of the PDGF receptor family, acts upstream of PRL-3 to promote invasion, and further suggest that PRL-3 may mediate some of the invasion and metastasis-associated functions of PDGFR, Src or related kinases.

## Methods

### Cell lines, plasmids, transfections and antibodies

SW480 colon adenocarcinoma cells and H1299 nonsmall cell lung cancer cells were obtained from the UNC-Lineberger Comprehensive Cancer Center Tissue Culture Facility. Src/Yes/Fyn-deficient (SYF) mouse embryo fibroblast (MEF) cells [26] were a gift from Ken Jacobson (UNC). SW480 cells were maintained in 0% CO_2_ in Leibovitz L15 medium (with glutamine) supplemented with 10% fetal calf serum. SYF and H1299 cells were maintained in 10% CO_2_ in Dulbecco's modified Eagle medium with high glucose (DMEM-H) supplemented with 10% fetal calf serum. To generate mammalian expression plasmids (pSBP-PRL-3) encoding fusion proteins that could be pulled down using streptavidin-Sepharose (GE Healthcare, Uppsala, Sweden), coding sequences for PRL-3 and mutants were cloned into pSBP, a pcDNA3-based vector into which we had previously inserted streptavidin-binding protein (SBP). All transfections were performed on 50–60% confluent cells plated in 100 mm dishes, with 1 µg each of the indicated plasmid(s), using the transfection reagent TransIT-LT1 (Mirus) according to the manufacturer's protocol.

For bacterial expression, PRL-3 or mutants were cloned into pGEX2T (GE Healthcare) fused to glutathione-S-transferase (GST). To generate SW480 cells stably expressing PRL-3 or mutants, coding sequences were cloned into pBABE-HAII. pUSE-Src was obtained from Upstate Biotechnology (Lake Placid, NY). Western blot analyses were done with the following antibodies: anti-PRL-3 (P-0498, Sigma, St. Louis, MO), pY99 anti-phosphotyrosine (Santa Cruz Biotechnology, Santa Cruz, CA), anti-Src antibody (32G6) and anti-phospho-Src family Tyr 416 antibody (#2101, Cell Signaling Technologies, Danvers, MA) and anti-RhoC (a kind gift from K. Van Golen, University of Delaware) [27].

### Endogenous PRL-3 immunoprecipitation and treatment with platelet-derived growth factor (PDGF)

To evaluate endogenous PRL-3 phosphorylation, cells were treated for 1 h with 100 mM pervanadate. Pervanadate was made fresh by adding 1 µL of 30% hydrogen peroxide to 200 µL of 50 mM sodium orthovanadate in water. Following treatment, cells were lysed in RIPA (50 mM Tris, pH 8.0, 150 mM sodium chloride, 1% Nonidet P-40 substitute, 0.5% deoxycholate, 0.1% sodium dodecylsulfate) +100 mM pervanadate and protease inhibitors. PRL-3 antibody (Sigma-Aldrich, St. Louis, MO) was diluted 1:250 in lysates containing 200-500 µg total protein. Samples were incubated at 4°C O/N with rocking. Immune complexes were precipitated with protein A/G Sepharose, washed three times in RIPA, and resuspended in protein gel sample buffer without reducing agents prior to SDS-PAGE and western blot analysis. For treatment with PDGF, SW480 cells were starved overnight in the absence of serum. Cells were then treated with the Src inhibitor SU6656 (1, 5, or 10 µM; Calbiochem, San Diego, CA)) or with vehicle (DMSO) for 1 h. Alternatively, Src expression was ablated using siRNA as described below, and serum starved overnight prior to PDGF treatment. Cells were then treated for 20 min with human PDGF-BB (Sigma, 20 ng/mL) and lysed for PRL-3 immunoprecipitation as described above.

### siRNA-mediated knockdown of Src

Expression of endogenous Src was reduced using an siRNA pool containing four targeting sequences (Dharmacon/Thermo Fisher Scientific, Rockford, IL). SW480 cells were transfected according to the manufacturer's protocol using a final siRNA concentration of 50 nM. The Dharmacon ON-TARGET*plus* non-targeting siRNA pool was used as a negative control. Cells were maintained in standard conditions for five days and treated with pervanadate for 1 h prior to lysis and PRL-3 immunopreciptiation as described above.

### Exogenous PRL-3 pull downs

Following treatment, cells were lysed in streptavidin lysis buffer (SLB: 10 mM Tris, pH 8.0, 150 mM sodium chloride, 0.1% NP-40). To pull down and detect SBP-tagged PRL-3, streptavidin-Sepharose beads were incubated with clarified lysates for 1 h at 4°C with rocking, washed three times with SLB and subjected to SDS-PAGE and western blot analysis using anti-PRL-3 antibody. For inhibitor studies, the Src family kinase inhibitor SU6656 was added to the culture medium 1 h prior to pervanadate treatment.

### *In vitro* Src kinase assays

GST-tagged PRL-3 was induced from the pGEX2T vector in *E. coli* bacteria, purified on glutathione beads, and subjected to *in vitro* phosphorylation by purified p60 c-Src (14–117, Millipore, Inc., Billerica, MA). The *in vitro* kinase assay was carried out with ∼500 ng of each protein according to the manufacturer's protocol, except that non-radiolabelled ATP was used as the phosphate donor. Samples were then subjected to SDS-PAGE and western blot analysis for phosphotyrosine and for PRL-3.

### Matrigel invasion assays

The invasiveness of SW480 cells stably expressing PRL-3 or a phosphodeficient mutant (PRL-3 Y53F) was evaluated using Matrigel invasion chambers (BD Biosciences, Bedford, MA) as described previously [4]. SW480 cells (5×10^4^/well) were placed in the top section of the invasion chamber in the absence of serum. Medium containing 10% serum was placed in the bottom chamber to create a chemoattractant gradient. After 72 h invaded cells were stained and counted. For inhibitor studies, SU6656 was placed in both the top and bottom chambers. Data were analyzed using a two-tailed Student's *t*-test.

### Single cell motility assays

The effect of PRL-3 and the phospho-deficient mutant Y53F on cell motility was evaluated using a Nikon BioStation IM single cell recorder. H1299 cells (7×10^3^ cells/plate), whose basal adhesive and motile characteristics are suitable for this assay system, were plated on glass-bottom plates (MatTek, Ashlan, MA) and transfected with EGFP-tagged PRL-3, PRL-3 (Y53F) or empty vector. Time-lapse images of fluorescent cells were taken at 5 min intervals over 2 h. Average velocity of individual cells was evaluated using ImageJ (http://rsb.info.nih.gov/ij/). Non-fluorescent cells were also evaluated as controls. Data were analyzed using a two-tailed Student's *t*-test.

### RhoC activity assays

Levels of active RhoC-GTP were determined as previously described [4]. Briefly, SW480 cells stably expressing PRL-3 or mutants were starved in 0% serum overnight prior to lysis. Lysates containing 500-750 µg total protein were incubated at 4°C for 1 h with glutathione agarose beads carrying the GST-tagged Rho-binding domain of the RhoC effector, Rhotekin (GST-RBD, ∼100 µg). The beads were pelleted and subjected to western blot analysis for RhoC. For inhibitor studies, SU6656 was added 1 h prior to lysis.

## Results

### PRL-3 is phosphoryated *in vitro* by the Src tyrosine kinase, and endogenous PRL-3 is tyrosine-phosphorylated in cells

**Figure 1:**
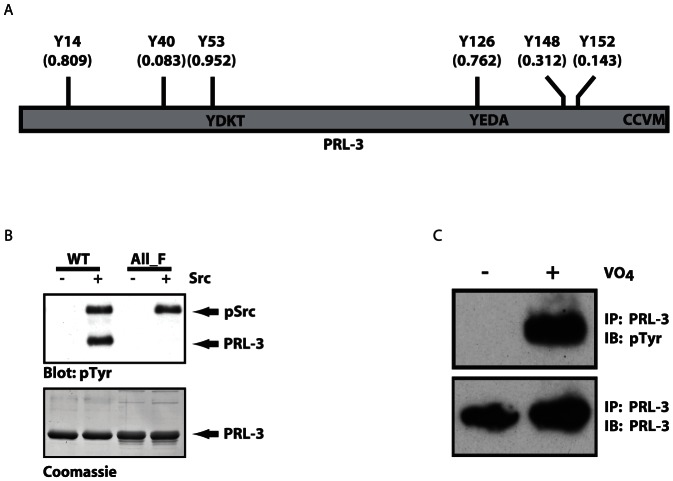
PRL-3 is phosphoryated in vitro by the Src tyrosine kinase, and endogenous PRL-3 is tyrosine-phosphorylated in cells. A) Schematic diagram of PRL-3 showing the relative positions of the six tyrosines evaluated in these studies, as well as the C-terminal CaaX motif. The numbers shown reflect the probability of phosphorylation at each site based on predictions by NetPhos 2.0. B) PRL-3 (“WT”) or a mutant in which all six tyrosines were substituted by phenylalanine (“All_F”) was fused to GST, purified from bacteria, and subjected to *in vitro* phosphorylation with purified Src. WT PRL-3 was phosphorylated by Src while the “All_F” PRL-3 mutant was not. Src itself is visible on the phosphotyrosine blot because it becomes autophosphorylated on Y416 (pSrc). Coomassie staining demonstrates equal amounts of PRL-3 protein in each reaction. C) To determine whether endogenous PRL-3 is also tyrosine phosphorylated in cells, SW480 cells were treated with the tyrosine phosphatase inhibitor pervanadate (VO_4_, 100 µM, 1 h) to enhance detection of transient tyrosine phosphorylation events. Endogenous PRL-3 was immunoprecipitated using anti-PRL-3 antibody, followed by SDS-PAGE and western blot analysis for either PRL-3 or phosphotyrosine.

Many cellular proteins, including some tyrosine phosphatases, are regulated by phosphorylation on tyrosine residues [14]–[21]. The tyrosine phosphatase PRL-1, which shares all six tyrosine residues in common with its close relative, PRL-3, has been shown to be phosphorylated *in vitro* by the tyrosine kinase Src [25], although the study did not determine the site of phosphorylation or whether it occurs *in vivo*. Analysis of PRL-3 using Netphos 2.0 (http://www.cbs.dtu.dk/services/NetPhos/) indicated a high probability of PRL-3 phosphorylation on tyrosines (Figure 1A). To determine whether Src can phosphorylate PRL-3 as it does PRL-1, we first performed an *in vitro* kinase assay in which bacterially expressed PRL-3 fused to glutathione-S-transferase (GST-PRL-3) was subjected to phosphorylation by purified Src. Using western blot analysis with anti-phosphotyrosine antibody, we observed that WT PRL-3 was phosphorylated on tyrosine in the presence of Src, whereas a mutant form of PRL-3 lacking all six tyrosines (designated “All_F”) was not (Figure 1B). We obtained identical results when the kinase assay was performed using ^32^P-ATP and phosphorylation was evaluated by autoradiography (data not shown). In neither case was GST alone phosphorylated (data not shown). Thus, PRL-3, like PRL-1, can be a direct substrate of Src tyrosine kinase activity *in vitro*.

We next wished to determine whether PRL-3 is also tyrosine phosphorylated in cells. We have shown previously that PRL-3 promotes motility and invasion in SW480 colon adenocarcinoma cells[4], properties that are also regulated by Src. Therefore, we treated SW480 cells, which express PRL-3 endogenously, with the tyrosine phosphatase inhibitor pervanadate to prevent dephosphorylation and thereby to promote the accumulation of phosphorylated PRL-3. Following immunoprecipitation using an anti-PRL-3 antibody, we performed western blot analysis for phosphotyrosine. Figure 1C shows that endogenous PRL-3 can be phosphorylated on tyrosine. The lack of detectable basal tyrosine phosphorylation in the absence of pervanadate suggests that this phosphorylation event is transient, evident only when the cellular phosphatase(s) responsible for dephosphorylation are blocked.

### Src is required for PRL-3 tyrosine phosphorylation

**Figure 2:**
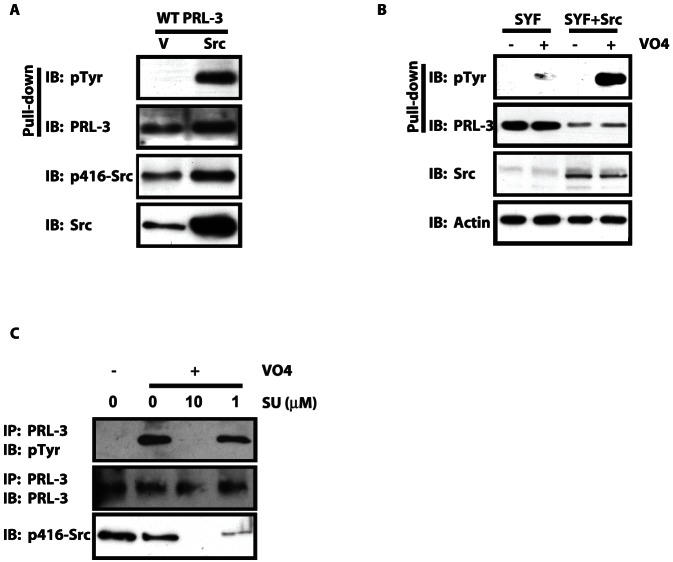
Src is required for PRL-3 tyrosine phosphorylation in cells. A) SW480 cells were co-transfected with both WT PRL-3 tagged with streptavidin binding protein (SBP) and WT Src in the absence of pervanadate, and then analyzed for phosphotyrosine by western blot following pull down with streptavidin beads. Src kinase activity was evaluated by analysis of the Src autophosphorylation site, Y416. B) WT PRL-3 was transfected into mouse embryo fibroblasts in which expression of Src, Yes and Fyn was simultaneously ablated genetically (SYF cells) or into SYF cells into which only Src had been reintroduced (SYF+Src). Cells were treated with pervanadate (VO_4_) and the phosphorylation of PRL-3 on tyrosines was then analyzed by western blot following pull down of PRL-3. C) SW480 cells were treated with the Src family kinase inhibitor SU6656 (10 µM or 1 µM, 1 h) prior to treatment with pervanadate and western blot analysis for PRL-3 and phosphotyrosine.

Src regulates some tyrosine phosphatases *in vivo*, either directly or indirectly [14], [17]. Having shown that PRL-3 can be a direct substrate of Src tyrosine kinase activity *in vitro* and that endogenous PRL-3 is tyrosine phosphorylated *in vivo*, we wished to determine the role of Src in modulating PRL-3 phosphorylation. First, to determine whether Src is sufficient to cause an increase in PRL-3 tyrosine phosphorylation, we co-transfected SW480 cells with both PRL-3 and Src, then probed for tyrosine phosphorylation of PRL-3 by western blot analysis. Figure 2A shows that, even in the absence of pervanadate-mediated inhibition of dephosphorylation, tyrosine phosphorylation of PRL-3 was easily detectable in the presence of Src but not with empty vector alone (V). Although these results do not indicate whether PRL-3 is a direct or indirect target of Src, and indeed unequivocal demonstration of a direct relationship between kinase and substrate in cells is technically not feasible with currently available technology, they do indicate that Src can act upstream of PRL-3 and is sufficient to mediate its tyrosine phosphorylation in cells. Of course, these results do not rule out a role for other tyrosine kinases in modulating PRL-3 function, whether in addition to or in place of Src itself.

To confirm the importance of Src as a modulator of PRL-3 phosphorylation, we next performed a complementary experiment in which we transfected PRL-3 into SYF cells, which are MEFs that are simultaneously deficient in expression both of Src and of the related Src family kinases Yes and Fyn [26]. We then treated these cells with the phosphatase inhibitor pervanadate to reveal phosphorylated PRL-3. SYF cells displayed a low level of PRL-3 tyrosine phosphorylation, which was greatly increased by the stable reintroduction of Src (SYF+Src) (Figure 2B). These results confirm that Src itself is capable of promoting the phosphorylation of PRL-3. It is not clear whether the reduced levels of total exogenous PRL-3 protein seen consistently in SYF+Src compared to SYF cells result from effects of Src on PRL-3 expression, stability or some other factor. However, PRL-3 expression does not correlate with PRL-3 phosphorylation (compare Figure 2B, lanes 1 and 2, or lanes 3 and 4), indicating that phosphorylation is unlikely to affect protein stability, for example. Alternatively, Src expression in SYF+Src cells might decrease transcription of exogenous PRL-3 message, although it is not clear how Src activity would affect transcription driven by the strong cytomegalovirus (CMV) promoter in the PRL-3 expression plasmid. We conclude that this observation may be an artifact of the experimental conditions.

We observed that vanadate was not required to detect phosphorylation of PRL-3 by Src in SW480 cells (Figure 2A) whereas it was required in SYF cells (Figure 2B). We speculate that SW480 cells, which are derived from a primary colon tumor and express a transformed phenotype, activate WT Src more effectively than untransformed, MEF-derived SYF cells, and thereby induce basal levels of Src activity and PRL-3 phosphorylation sufficient to detect without inhibition of dephosphorylation.

To determine whether Src is necessary for PRL-3 tyrosine phosphorylation, we pretreated SW480 cells with the Src family kinase (SFK) inhibitor SU6656 (10 µM or 1 µM, 1 h), followed by treatment with pervanadate to reveal phosphorylation of endogenous PRL-3 as done for Figure 1B. Whereas pervanadate caused accumulation of phospho-PRL-3 in the presence of vehicle only, SU6656 inhibited PRL-3 phosphorylation in a dose-dependent manner, such that 1 µM inhibited phosphorylation by ∼20% and no phospho-PRL-3 was detected in the presence of 10 µM of this SFK inhibitor (Figure 2C). This indicates that phosphorylation of endogenous PRL-3 requires the activity of at least one member of the Src family of tyrosine kinases, and supports a model in which an SFK acts upstream of PRL-3 to cause its phosphorylation *in vivo*. Together, these observations demonstrate that Src is both sufficient and necessary to promote phosphorylation of endogenous PRL-3 in SW480 cells, although they do not rule out the possibility that other kinases may also serve this role in other contexts. Indeed, the presence of weak but detectable PRL-3 tyrosine phosphorylation in SYF cells (Figure 2B) suggests that kinases other than Src, Yes or Fyn can also play this role to some extent.

### PDGF stimulates PRL-3 phosphorylation in a Src-dependent manner

**Figure 3:**
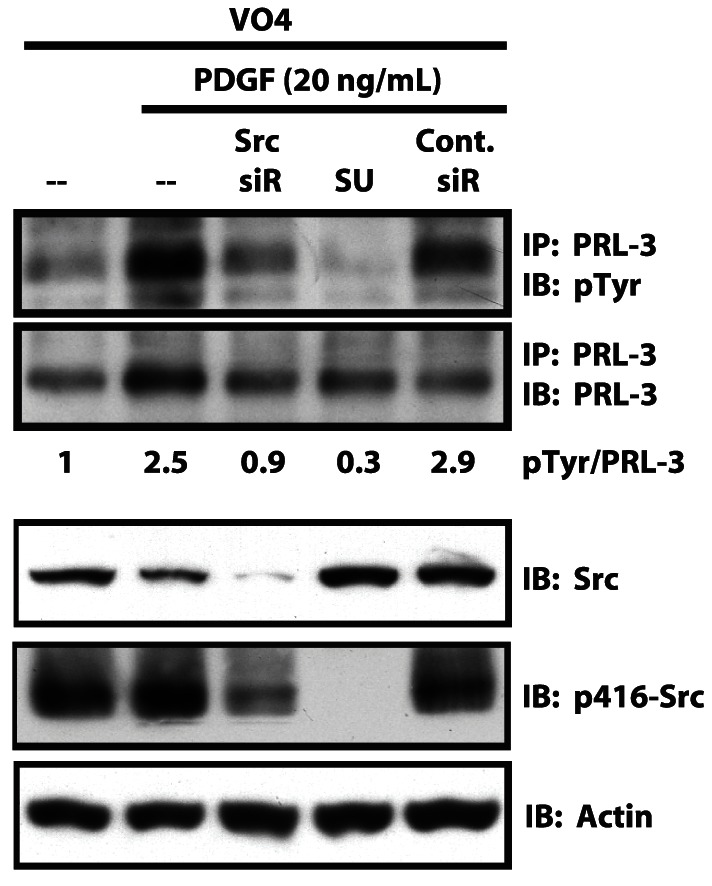
PDGF stimulates PRL-3 phosphorylation in a Src-dependent manner. Phosphorylation of endogenous PRL-3 in SW480 cells was evaluated as in Figure 1B following PDGF stimulation (20 ng/mL, 20 min). siRNA was used to reduce Src expression (“Src siR”), or the Src inhibitor SU6656 (5 µM) was used to inhibit Src activity (“SU”). Phosphotyrosine on PRL-3 was analyzed five days after siRNA transfection, and the degree of phosphorylation compared to that seen with control nontargeted siRNA (“Cont. siR”).

To determine whether PRL-3 could be phosphorylated in response to a physiological stimulus, we treated SW480 cells with platelet-derived growth factor (PDGF), the receptors for which contribute to aspects of tumor development [28]-[30]. Figure 3 shows a 2.5-fold increase in endogenous PRL-3 phosphorylation in response to PDGF, suggesting that PRL-3 may constitute an important downstream mediator of PDGF signaling. Further, ablation of Src expression using siRNA or inhibition of Src activity using the Src inhibitor SU6656 (5 µM, 1 h) blocked PDGF-induced PRL-3 phosphorylation, supporting a model in which PDGF induces the phosphorylation of PRL-3 via Src. Autophosphorylation of Src at tyrosine 416 was evaluated as a marker of Src kinase activity.

### Residue 53 is the major site of PRL-3 tyrosine phosphorylation

To identify the site(s) of tyrosine phosphorylation on PRL-3, we first analyzed the PRL-3 sequence using NetPhos 2.0, which predicted high probabilities for phosphorylation on tyrosine residues 14, 53, and 126 (Y14, Y53, Y126, Figure 1A). Based on this analysis we generated point mutants of PRL-3 in which tyrosines 14, 53 or 126 were mutated individually to non-phosphorylatable phenylalanines (Y14F, Y53F or Y126F, respectively). Each of these mutants, as well as WT and All_F, was then expressed in SW480 cells. After pervanadate treatment, PRL-3 proteins, which were also tagged with streptavidin-binding protein (SBP), were precipitated using streptavidin-Sepharose and then immunoblotted for total PRL-3 or for phosphotyrosine.

**Figure 4:**
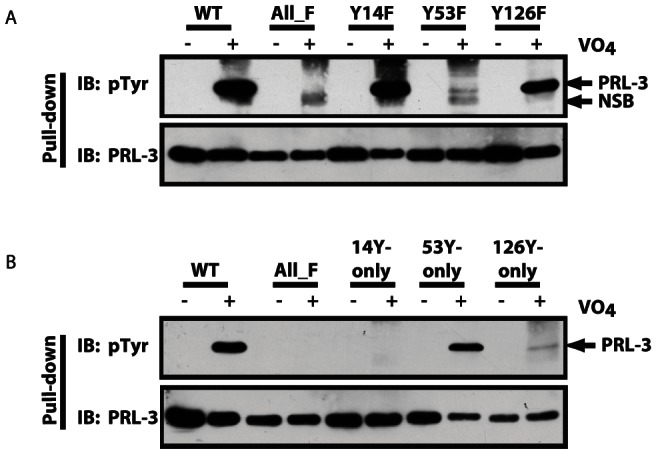
Residue 53 is the primary site of PRL-3 tyrosine phosphorylation. A) To identify the sites of phosphorylation, SW480 cells were transfected with expression constructs encoding wild-type PRL-3 or mutants in which all six or specific individual tyrosines were mutated to nonphosphorylatable phenylalanines (All_F, Y14F, Y53F, Y126F). Each was expressed as a protein fused to streptavidin-binding protein (SBP). Following pervanadate treatment, SBP-tagged PRL-3 was precipitated using streptavidin-Sepharose, and analyzed by SDS-PAGE and western blot for either PRL-3 or phosphotyrosine. A non-specific band (NSB) appears in all VO_4_-treated lanes at slightly a faster mobility than PRL-3. B) Conversely, PRL-3 mutants in which all but one tyrosine was mutated to phenylalanine, and which therefore contain a single tyrosine only at position 14 (14Y-only), 53 (53Y-only) or 126 (126Y-only), were analyzed similarly.

Similar to endogenous PRL-3, tyrosine phosphorylation of exogenous PRL-3 was also revealed by pervanadate treatment (Figure 4A). As seen previously, the All_F mutant was not phosphorylated. The band appearing in the All_F + pervanadate lane is a non-specific band (NSB), which appears in all lanes at a slightly faster mobility than PRL-3, and can be clearly distinguished from phospho-PRL-3 (see Y53F lane). Mutation of Y53 almost completely prevented the phosphorylation of PRL-3 (Figure 4A), indicating that this residue is the major site for this modification. The small amount of residual phosphorylation indicates that at least one other site can also be phosphorylated, albeit to a much lesser extent. Mutation of Y126 reduced overall phosphorylation marginally, indicating that this residue can be phosphorylated but is not the preferred site. Y126 may account for the residual phosphorylation seen with the Y53F mutant. Mutation of Y14 had no detectable effect on PRL-3 phosphorylation, thereby ruling out this site as a major target for tyrosine phosphorylation *in vivo*.

To confirm these observations, we generated a complementary set of PRL-3 mutants in which only a single tyrosine was present in each, at residues 14, 53 or 126, while the remaining five tyrosines were mutated to phenylalanines (14Y-only, 53Y-only or 126Y-only). As shown in Figure 4B, 53Y-only supported phosphorylation comparable to that seen with WT PRL-3, a finding consistent with this site as the major site of phosphorylation, and with the nearly complete loss of phosphorylation of the Y53F mutant shown in Figure 4A. Similarly, a small amount of phosphorylation was detectable with the 126Y-only mutant, consistent with the data shown in Figure 4A. Collectively, these observations demonstrate that endogenous PRL-3 can be phosphorylated on tyrosines *in vivo*, and that phosphorylation likely occurs almost exclusively on Y53 and to a far smaller extent on Y126. Based on these results, we focused our functional analyses on PRL-3 Y53F.

### PRL-3 promotion of invasion and cell motility requires Y53

**Figure 5:**
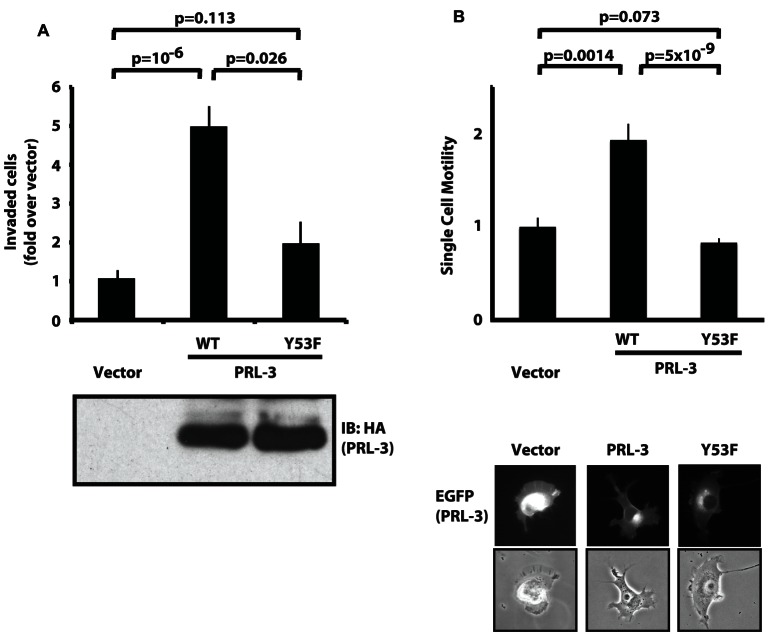
PRL-3 promotion of invasion and cell motility requires an intact Y53. A) SW480 cells stably expressing WT PRL-3 (WT) or a phosphorylation-deficient mutant lacking tyrosine 53 (Y53F) were subjected to Matrigel invasion assays. Data from three independent assays are shown. For each sample, 5x10^4^ cells were placed in the top chamber in the absence of serum; the bottom chamber contained medium supplemented with 10% serum. After 72 h, invaded cells were fixed, stained and counted. Equal expression of WT HA-tagged PRL-3 and the Y53F mutant was confirmed by HA blot. B) H1299 cells were transiently transfected with EGFP-tagged PRL-3, the Y53F mutant or vector only. The motility rate of individual cells was evaluated using a BioStation IM live cell recorder. Data shown are from three independent assays, and include a total of at least 35 cells for each sample. Images of representative cells expressing each EGFP-tagged protein are shown below the graph. All images were captured under identical microscopic and photographic settings. Data in both A and B are shown +/− SEM, and *p*-values were calculated using a two-tailed Student's *t*-test.

Our results show that Src kinase activity leads to the tyrosine phosphorylation of PRL-3, primarily on Y53. Both PRL-3 and Src play well-established roles in cell motility, and both are implicated by numerous correlative and functional studies in tumor invasion and metastasis [1]-[3], [31]. This suggests that Src may regulate the invasion- and metastasis-associated functions of PRL-3 through phosphorylation. To begin to address this possibility, we first determined whether phosphorylation of PRL-3 affects its ability to promote invasion. SW480 cells stably expressing wild-type PRL-3 or a mutant lacking tyrosine 53 (Y53F) were evaluated for their ability to invade through Matrigel. Figure 5A shows that wild-type PRL-3 promoted a 4-5-fold increase in the invasiveness of SW480 cells, whereas the Y53F mutant promoted an increase of only 40% that of WT, indicating that phosphorylation at this site is likely required for full PRL-3-induced invasion. In each assay, at least 20 control cells invaded.

We next determined whether the phosphorylation-deficient mutant of PRL-3 was still able to promote cell motility, which is a necessary component of the invasive phenotype. Because SW480 cells are not suitable for this type of analysis, we used H1299 cells, which have suitable properties of basal adhesiveness and motility. Cells were transiently transfected with EGFP-tagged PRL-3 or the Y53F mutant. The average velocities of individual cells were measured and compared to the velocities of cells expressing EGFP only. Figure 5B shows that wild-type PRL-3 significantly increased the average speed of H1299 cells by two fold, whereas the Y53F mutant had no significant effect. Together with the data shown in Figure 5A, these results confirm that PRL-3 promotes both invasion and motility, and that tyrosine 53 is crucial to both phenotypes, presumably due to a requirement for the phosphorylation event that occurs at this site.

### PRL-3 requires the activity of a Src family kinase to promote invasion and RhoC activation

**Figure 6:**
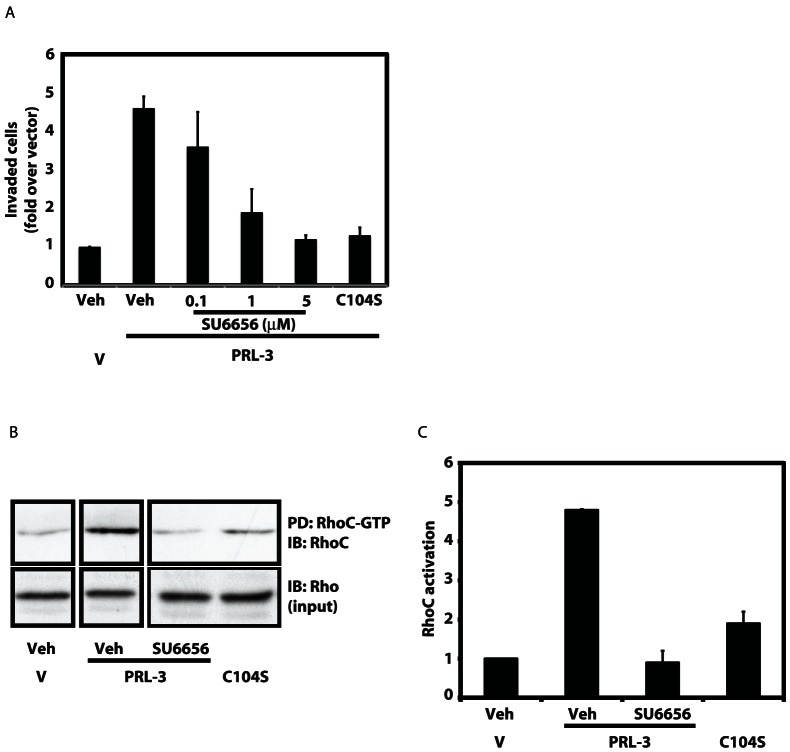
PRL-3 requires the activity of a Src family kinase to promote invasion and RhoC activation. A) SW480 cells expressing WT PRL-3 were subjected to Matrigel invasion analysis as in Figure 5. The Src family kinase inhibitor SU6656 was added to both the top and bottom chambers at the indicated concentrations. Cells expressing phosphatase-inactive PRL-3 (C104S) served as a negative control. Data are shown normalized to basal levels of invasion of vector-expressing cells. Results from four assays are shown +/− SD. B) SW480 cells expressing WT PRL-3 were treated with the Src inhibitor, SU6656, and analyzed for levels of active, GTP-bound RhoC using a Rhotekin-RBD pull down assay. Images from a representative assay are shown. Lanes containing samples not relevant to this work have been removed. All panels were taken from the same assay using the same photographic exposure time. Phosphatase-inactive PRL-3 (C104S) served as a negative control. C) Data quantitated from at least three pull down assays as in panel B are shown +/− SEM.

Our data show that PRL-3 is phosphorylated downstream of Src (Figures 1, 2 and 3), that Y53 is the major site of tyrosine phosphorylation (Figure 4) and that Y53 is required for full promotion of invasion (Figure 5A), presumably because Y53 must be phosphorylated. These observations predict that PRL-3 requires Src activity to promote invasion and possibly other functions. To test this hypothesis, we next determined whether inhibition of Src activity affected the ability of PRL-3 to promote invasion. The ability of SW480 cells stably expressing wild-type PRL-3 to invade through Matrigel was evaluated in the presence or absence of the Src family kinase inhibitor SU6656. PRL-3-induced Matrigel invasion was blocked by SU6656 in a dose-dependent manner, with 90% inhibition seen at 5 μM (Figure 6A). At this concentration, invasion was reduced to a level comparable to that seen with phosphatase-inactive PRL-3 C104S or empty vector. We have shown previously that PRL-3 promotes activation of the small GTPase RhoC [4], which contributes to tumor cell motility and invasion through regulation of the actin cytoskeleton [32]-[34]. Figure 6B shows a representative RhoC-GTP assay in which active RhoC-GTP was selectively pulled down from cell lysates and quantitated by western blot analysis. Data from at least three independent pull down assays are summarized in Figure 6C, which shows that inhibition of Src family kinases with SU6656 blocked PRL-3-induced RhoC activation, reducing it from 5-fold to basal levels. Together these observations indicate that tyrosine phosphorylation and Src activity are both required for PRL-3-dependent biological functions, and that Src regulation of PRL-3 may be part of an important signaling pathway contributing to tumor invasion and metastasis.

## Discussion

Numerous correlative studies in tumors of the colon, stomach, breast, pancreas and ovaries have shown that PRL-3 expression increases with tumor progression (reviewed in [2], [35]). Further, PRL-3 expression also correlates with tumor clinicopathology and disease outcome in a variety of tumor types [36]–[40]. These correlative studies are supported by numerous functional studies (reviewed in [1], [3]). In cell culture and mouse model systems, ectopic expression of PRL-3 promotes cell motility and invasion [4], [41]–[45]. Conversely, silencing of endogenous PRL-3 by siRNA inhibits both invasion and metastasis in model systems [8], [46]. Further, PRL-3 knockdown mice develop significantly fewer colon tumors in response to azoxymethane than wild-type mice [47], directly demonstrating a role for PRL-3 in tumor formation *in vivo*. Collectively, these observations support a causal role for PRL-3 in tumor invasion and metastasis and have led to the frequent characterization of PRL-3 as a "metastasis-associated tyrosine phosphatase". Our data suggest that these biological functions of PRL-3 are regulated by a Src family tyrosine kinase, and that this may be due to Src-mediated phosphorylation of tyrosine 53, a residue that we show here is both the major site of tyrosine phosphorylation and a requirement for full PRL-3 activity. Specifically, we have shown that: 1) Src itself can phosphorylate PRL-3 directly *in vitro*, 2) endogenous PRL-3 is tyrosine phosphorylated, 3) Src is required for tyrosine phosphorylation of PRL-3, 4) PDGF can stimulate PRL-3 phosphorylation in a Src-dependent manner, 5) of the six tyrosines in PRL-3, Y53 is the major site of phosphorylation, 6) Y53 is required for PRL-3 promotion of motility and invasion, and 7) PRL-3 requires the activity of a Src family kinase to fully promote motility, invasion and RhoC activation. Collectively these results support a model in which Src causes phosphorylation of PRL-3 on Y53 to promote its pro-invasion functions, and suggest for the first time that the metastasis-associated tyrosine phosphatase PRL-3 may itself be regulated by post-translational modification.

There are precedents for regulation of tyrosine phosphatases by their own tyrosine phosphorylation. PTP1B, LMW-PTP and Shp-2 are each reported to be phosphorylated on tyrosines with functional consequences of that modification [16]-[22]. *In vitro* analyses suggest that phosphorylation may directly affect the catalytic activity of both LMW-PTP and PTP1B, although the structural changes that are presumably responsible for this are not clear. In the case of Shp-2, tyrosine phosphorylation is thought to induce the formation of intramolecular bonds between phosphotyrosines and internal Src homology-2 (SH2) domains, thus exposing the active site and increasing catalytic activity. Phosphorylation of LMW-PTP has also been shown potentially to create a binding site for the adapter protein Grb2 [17], suggesting a role for the phosphatase as a scaffolding protein in Ras signaling pathways.

It is not yet clear by what mechanism phosphorylation affects the function of PRL-3, although several possibilities exist. As with LMW-PTP, PTP1B and Shp-2, phosphorylation of PRL-3 at Y53, which lies in the second alpha-helical domain adjacent to the active site [48], may directly affect catalytic activity, or influence subcellular localization. Although we have not observed any obvious effects of phosphorylation on subcellular localization by confocal immunofluorescence microscopy (data not shown), it is possible that phosphorylation induces more subtle differences that are nevertheless functionally important, such as restriction to specific membrane-binding sites. As with LMW-PTP, phosphorylation of PRL-3 could also create new binding sites for proteins containing SH2 domains, such that PRL-3 may act as a membrane-associated scaffolding protein as well as a phosphatase. Further, given that tyrosine 53 is found in all three PRL proteins, and that the surrounding sequence is highly conserved, it will be of interest to determine whether a similar functional relationship exists between PRL-1 or PRL-2 and Src. It has also been shown recently that PRL-3 associates with and promotes the activity of the GTPase Arf1 at the Golgi [42], suggesting that PRL-3 may promote cell motility by affecting vesicular trafficking and protein secretion at the leading edge. Interestingly, both Golgi localization and Arf1 association of PRL-3 depend on the sequence MKYE [42], which includes tyrosine 126. Although we have focused here on tyrosine 53, we also detected phosphorylation of tyrosine 126 (Figure 4B), suggesting that PRL-3/Arf1 activities may be modulated by phosphorylation of this site. Future studies will address this issue.

A structural feature unique to PRL PTPs is that they form non-covalent trimers *in vitro* and *in vivo* [49], [50], suggesting that quaternary interactions may be required for full catalytic activity, and that disruption of this structure, possibly through phosphorylation, may affect function. However, it seems unlikely that the trimerization of PRL-3 is affected by phosphorylation at tyrosine 53, which is distant from the trimer interface, based on the highly homologous sequence of PRL-1 [49], [50]. Now that we have established for the first time that tyrosine phosphorylation is an important process affecting the biological functions of PRL-3, it will be important next to further define the structural and/or biochemical mechanisms that produce these effects.

There is an increasing appreciation that signal transduction occurs in the context of complex feedback loops rather than a simple unidirectional flow of linear pathways [51]-[53]. For example, EGF stimulation of EGFR activates the Ras>Raf>MEK pathway, which in turn regulates expression of both EGFR and its ligands [54]. Together with our observations here, previous reports supporting a functional connection between Src and PRL phosphatases also suggest a reciprocal relationship between these two proteins. Inhibition of PRL-1 expression has been shown to reduce expression of both Src and the important Src substrate, p130Cas [55], suggesting that PRL-1 transcriptionally activates key components of Src signaling pathways, and may promote both normal and aberrant Src-dependent phenotypes. Interestingly, PRL-3 inhibits expression of the Src regulatory protein c-Src kinase (Csk) [43], which normally blocks the activation of Src by phosphorylating it on tyrosine 528. Together these observations suggest that PRL PTPs may upregulate Src signaling by distinct but complementary mechanisms. Both models place PRL phosphatases upstream of Src expression and activity. In addition, our results suggest that Src may also act upstream of PRL-3, affecting PRL-3 functions through tyrosine phosphorylation. In addition, such a functional link suggests that PRL-3 and Src may cooperate in colon carcinoma, in which the expression of PRL-3 [2] as well as the expression and activity of Src [56], [57] all increase with advancing disease. If so, evaluation of expression of PRL-3 and Src together in colon tumors may help predict more aggressive disease and poor prognosis, and may identify patients who could benefit from more aggressive treatment options. We are currently addressing these questions, the results of which will be described elsewhere.

By functionally linking PRL-3 and Src, which individually play established roles in cell motility, invasion and metastasis, our model helps to better define the biochemical mechanisms of these important aspects of tumor progression. In light of recent progress towards clinically-relevant Src inhibitors (reviewed in [31], [58]) and increasing effort devoted to identifying and characterizing inhibitors of PRL-3 (reviewed in [2]), our work offers new insights into the development and clinical application of molecularly targeted cancer therapeutics. Our results suggest that PRL-3 may mediate some tumorigenic functions of Src, particularly those associated with control of small GTPases, such as RhoC, and tumor cell motility and invasion. If so, PRL-3 expression, which increases with progression in many tumor types, may help identify tumors that will be sensitive to Src inhibitors. Similarly, Src expression may reflect sensitivity to PRL-3 inhibitors when sufficiently selective and potent ones become available. In this regard, it would be interesting to determine whether expression levels of Src and PRL-3 correlate in tumors, suggesting a general upregulation of the Src > PRL-3 signaling axis that we have proposed here, and whether such a correlation has implications for the aggressiveness of the disease, or patient outcome. We are working to address this possibility.
